# Fifth decennium after the arterial switch operation for transposition of the great arteries

**DOI:** 10.1016/j.ijcchd.2023.100451

**Published:** 2023-03-23

**Authors:** Sebastiaan W.H. van Wijk, Maaike Wulfse, Mieke M.P. Driessen, Martijn G. Slieker, Pieter A. Doevendans, Paul H. Schoof, Gert Jan J. Sieswerda, Johannes M.P.J. Breur

**Affiliations:** a Department of Pediatric Cardiology, Wilhelmina Children's Hospital, Utrecht, the Netherlands; b Department of Cardiology, Radboud UMC Nijmegen, Nijmegen, the Netherlands; c Department of Cardiology, Division of Heart and Lungs, University Medical Centre Utrecht, University of Utrecht, Utrecht, Netherlands; d Netherlands Heart Institute, Utrecht, Netherlands; e Central Military Hospital, Utrecht, Netherlands; f Paediatric Cardiothoracic Surgery, Wilhelmina Children's Hospital, University Medical Centre Utrecht, the Netherlands

**Keywords:** Transposition of the great arteries (TGA), Arterial switch operation (ASO), Follow-up, Echocardiography, Aortic valve, Long-term

## Abstract

**Background:**

From 1977 onwards, patients with both simple and complex transposition of the great arteries (TGA) have been treated with the arterial switch operation (ASO) in the Wilhelmina Children's Hospital/University Medical Center Utrecht the Netherlands. In this study, we compared mortality and morbidity between two patient groups: A. operated before and B. after 1991, specifically focusing on late ventricular function and reinterventions.

**Methods:**

A single institution retrospective cohort study was performed on patients who had an ASO for either simple or complex TGA. Data were collected from medical records. The entire patient cohort (n = 283) was divided in a group with more than 30 years of follow-up (A) and a group with less than 30 years of follow-up (B). Clinical and standardized echocardiographic follow-up was evaluated.

**Results:**

Group A consisted of 79 patients, of whom follow-up was available in 59 patients (median follow-up 34.8 years, IQR 33.0–36.9). Group B consisted of 204 patients, of whom 195 long-term survivors (median follow-up 14.9 years, IQR 10.0–21.2). Early survival was best in group B (A: 67.8% vs. B: 96.6%, p < 0.001), whereas late mortality (in total 1.8%) was similar for both groups. Reinterventions, corrected for follow-up time, were more frequent in group A (p = 0.005). In total 65 patients (25.1%) required 105 late reinterventions including 4 late aortic valve replacements. The mode of reinterventions has shifted over time, from surgical to more catheter-based (p = 0.03). The vast majority of patients functioned in NYHA class I. In contrast to the recent cohort, who have a normal average LVEF (%), the average LVEF in the oldest cohort was in the bottom percentile of normal range.

**Conclusion:**

The majority of patients in their fifth decade after ASO are in functional class I. Early outcome improved showing reduced mortality and need for reoperation. However, a trend towards reduced left ventricular function and late aortic valve replacements justify further research.

## 1 Introduction

The arterial switch operation (ASO) has become the standard treatment for simple and complex transposition of the great arteries (TGA) since late 1980's [1]. With improved operative survival, focus in outcome studies has shifted to long-term morbidity including residual lesions such as pulmonary artery stenosis, neo-aortic root dilation, neo-aortic regurgitation, coronary stenosis, arrhythmia [2,3], and quality of life [4]. Reinterventions for residual lesions are performed relatively frequently and range between 16 and 30% (within 25 years of follow-up) [[5], [6], [7]]. Information on outcome beyond 30 years is limited and focused on indications and risk factor prediction for reinterventions [8,9]. However, further insight into a time-related change in reintervention and late ventricular function is not reported.

Our ASO program started in 1977 and aimed to correct the patient's TGA along with all associated lesions in a single operation. The objective of this study was to compare differences in mortality and morbidity between patients operated on in the earliest era with those operated more recently, with a specific focus on late reinterventions and ventricular function change.

## 2 Methods

### 2.1 Study populations

A single institution (Wilhelmina Children's Hospital Utrecht, the Netherlands) retrospective cohort study was performed on patients who underwent the ASO for TGA between January 1977 and December 2014 [10]. Patients were split in two cohorts depending on the date of surgery: group A (1977–1986) and group B (1987–2014). Children (age <18 years) were included to display trends in reinterventions and complications.

Data were collected from review of medical records. The minimum follow-up duration was 6 years. The local Medical Ethics Committee approved a waiver of consent.

Anatomical subtypes were categorized as simple TGA (with intact ventricular septum; IVS), complex TGA (with associated congenital heart lesions such as ventricular septal defect and/or arch anomaly) [11]. Associated lesions were classified as aortic arch anomlies (coarctation of the aorta; interrupted aortic arch), valvular defects (bicuspid aortic valve, straddling valves), left or right outflow tract obstruction (LVOTO; RVOTO).

### 2.2 Data collection and analysis

Details on concomitant cardiac anomalies, surgical techniques and early postoperative outcome were gathered from patient records (Table 1). Operative mortality was defined as all deaths, regardless of cause, occurring during the hospitalization in which the operation was performed, up to 30 days (including patients transferred to other acute care facilities); and all deaths, regardless of cause, occurring after discharge from the hospital but before the end of the 30th postoperative day [12,13].

**Table 1:** Patient characteristics per group.

	Total	Group A, follow-up ≥ 30 years	Group B, follow-up < 30 years	p-value
N	283	79	204	
Median follow-up, years (min-max)	19.1 (6.6–43.5)	34.8 (30.6–43.5)	14.9 (6.6–29.6)	
Age at ASO, days (IQR)	9 (7–26)	57 (7–184)	8 (6.3–13)	**<0.001**
Female, N (%)	78 (27.6)	22 (27.8)	56 (27.5)	0.95
Perioperative complication, N (%)	48 (17.0)	6 (7.6)	42 (20.6)	**0.009**
Mortality, N (%)	29 (10.2)	20 (25.3)	9 (4.4)	**<0.001**
Operative	24 (8.5)	17 (21.5)	7 (3.4)	**<0.001**
Late	5 (1.8)	3 (3.8)	2 (1.0)	0.21
Simple TGA, N/n (%)	155/256 (60.5)	28/52 (53.8)	127/204 (62.3)	0.27
Complex TGA^b^, N/n (%)	101/256 (39.5)	24/52 (46.2)	77/204 (37.7)	0.27
Aortic arch anomaly, N/n (%)	21/256 (8.2)	6/52 (11.5)	15/204 (7.4)	0.33
PAB, N/n (%)	23/238 (9.7)	18/43 (41.9)	5/195 (2.6)	**<0.001**
BAS, N/n (%)	136/233 (58.7)	25/37 (67.6)	111/196 (56.6)	0.22
Lecompte, N/n (%)	241/259 (93.1)	45/55 (81.8)	196/204 (96.1)	**<0.001**
Common coronary pattern^a^, N/n (%)	161/225 (71.6)	24/30 (80)	137/195 (70.3)	0.27
NYHA class I, N/n (%)	249/254 (98.0)	55/59 (93.2)	194/195 (99.5)	**<0.001**
NYHA class II, N/n (%)	5/254 (2.0)	4/59 (6.8)	1/195 (0.5)	**<0.001**

Abbreviations: ASO – Arterial Switch Operation; IQR – Inter-quartile range; TGA – Transposition of the Great Arteries; n – number of patients in group with variable measurement; PAB – Pulmonary artery banding; BAS – Balloon atrial septostomy.

Bold refers to statistically significant p-values.

a 1LCx, 2R (Leiden classification).

b Complex TGA refers to additional associated congenital heart lesions aside from the TGA such as ventricular septal defect and/or arch anomlies [11].

Early postoperative complications were categorized according to previous definitions [14]: renal failure requiring temporary or permanent dialysis, neurological deficit persisting at discharge, atrioventricular block or arrhythmia requiring a permanent pacemaker, postoperative mechanical circulatory support, phrenic nerve injury, or any unplanned reintervention before discharge (Table 2).

**Table 2:** Early postoperative complications [12].

Complication	N (total = 48)
Renal replacement therapy	0
Neurological deficit	6
Permanent pacemaker	3
Mechanical circulatory support	2
Phrenic nerve injury	8
Unplanned reintervention	29

Reinterventions were defined as any surgical or catheter-based intervention for residual cardiac lesions.

### 2.3 Echocardiography

Standard echocardiographic follow-up was performed every second year by an experienced, certified cardiac sonographer and reviewed by a pediatric or Adult Congenital Heart Disease (ACHD) cardiologist (Table 3) according to guidelines [[15], [16], [17]]. Left ventricular (LV) ejection fraction (EF; biplane Simpson), LV end diastolic diameter (EDd), interventricular septal end systolic diameter (IVSd), tricuspid annular plane systolic excursion (TAPSE), mitral valve early diastolic velocity (MV E) divided by atrial contraction velocity A, (MV E/A ratio), MV septal E divided by septal mitral annular early diastolic velocity (E/e’ ratio), septal annular early diastolic velocity (septal e’), maximal aortic sinus diameter, and valvular regurgitation were collected at the last outpatient visit [18].

**Table 3:** Univariate analysis of risk factors for reintervention.

Risk factor	Univariate		
	HR	95% CI	p-value
Years since ASO	1.038	1.010–1.066	**0.007**
Age at ASO	1.003	1.001–1.004	**0.006**
Early postoperative complications	1.910	1.051–3.469	**0.03**
Complex TGA	1.714	1.032–2.847	**0.04**
Aortic arch anomaly	4.865	2.665–8.881	**<0.001**
Pulmonary artery banding	1.881	0.924–3.830	0.08
Lecompte maneuver	0.667	0.302–1.475	0.32
Common coronary pattern	0.919	0.477–1.773	0.80

Abbreviations: ASO – Arterial switch operation; TGA – Transposition of the great arteries.

Bold refers to statistically significant p-values.

For LVEDd, IVSd, TAPSE, E/A ratio, E/e’ ratio and maximal aortic sinus diameter, only patients aged 18 years or older were included to enable comparison of the outcome measures with the standardized values for adults. Z-scores for these outcome measures in children were noted but not compared. LVEF (biplane Simpson) was only included in standard outpatient clinical follow-up for adults.

### 2.4 Functional outcome

Clinical parameters were collected from patient records at last clinical follow-up, including NYHA class and prescription of heart failure medication.

### 2.5 Statistical analysis

Continuous variables were summarized by mean ± standard deviation for normally distributed data or median and interquartile range (IQR) for data that was not distributed normally. Differences in group averages were tested with student's t-test (normally distributed) or Mann-Whitney-U test (not normally distributed) for continuous data or Chi square for categorical data. Event-free survival was estimated by Kaplan-Meier method. Difference in event-free survival was analyzed by log-rank test. Determinants for reintervention were analyzed with a Cox proportional hazards regression model, using a backward stepwise regression algorithm with entry p-value = 0.05 and removal p-value = 0.10. All analyses were performed with excluding cases in whom key variables were missing.

Statistical analysis was performed using IBM SPSS (IBM Corp. Released 2019. IBM SPSS Statistics for Windows, Version 26.0. Armonk, NY: IBM Corp).

## 3 Results

### 3.1 Study populations

Our cohort included 283 patients who had a median follow-up of 19.1 (IQR 11.1–28.1) years. Seventy-nine patients were operated before 1991 (group A), of which 59 were long-term survivors, being in their fourth decade after operation (median follow-up 34.8 years, IQR 33.0–36.9). This subgroup was compared to 204 patients operated after 1990 (group B), with 195 survivors (median follow-up 14.9 years, IQR 10.0–21.2). Group and patient characteristics are both shown in Table 1. Of notice, more group A patients (18 vs 5) had a two-stage repair, with a late arterial switch (day 57 vs day 8, p < 0.001) preceded by pulmonary artery banding (41.9% vs 2.6%, p < 0.001). Follow-up time was measured as the last moment of routine clinical follow-up, resulting in no patients being lost to follow-up.

### 3.2 Mortality

Operative mortality was *significantly higher* in the early group A, being 21.5% versus 3.4% for group B, P = < 0.001). Five (3 + 2) patients (1.8%) died during follow-up.

In group A, late death happened in a 23-year-old patient who died of an unknown cause, one was attributed to documented arrhythmia at age 16 in a patient with poor LV function after perioperative myocardial infarction, and one death was attributed to ventricular failure associated with pulmonary hypertension at age ten, after an uncomplicated operation.

In Group B, one death was attributed to a *S. aureus* septic shock at age 14 and one infant died at age 6 months of an unknown cause.

### 3.3 Early major postoperative complications

Of 283 patients, 48 (17.0%) had early major postoperative complications (Table 2). The unplanned reinterventions consisted of re-explorations for bleeding mainly (N = 19).

### 3.4 Reinterventions at follow-up

**Fig. 1:**
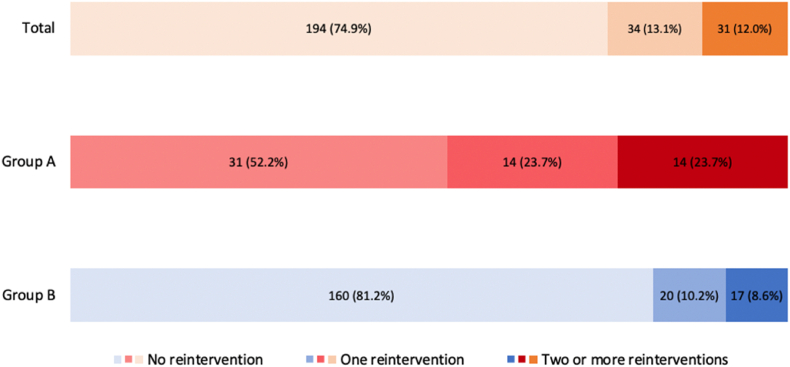
Late reinterventions in arterial switch operation patients (n = 259), subdivided for group A (follow-up ≥ 30 years) and group B (follow-up < 30 years).

**Fig. 2:**
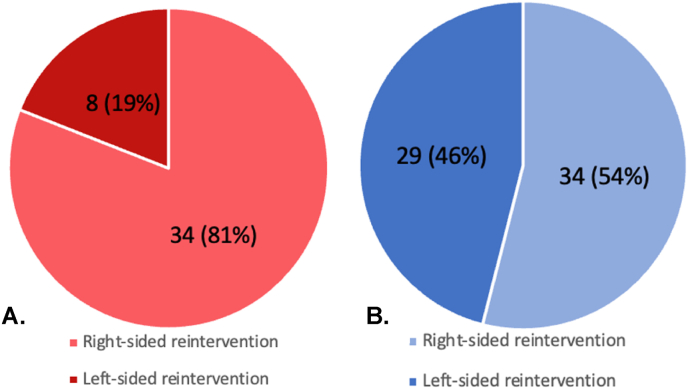
Right-versus left-sided late reinterventions in arterial switch operation patient. A: late reinterventions in group A (follow-up ≥ 30 years). B. late reinterventions in group B (follow-up < 30 years).

**Fig. 3:**
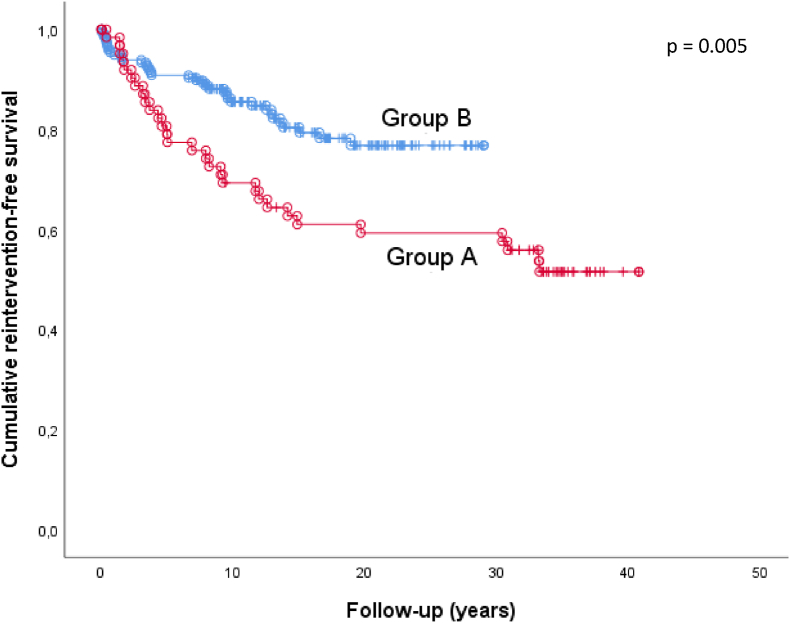
Kaplan-Meier plot of cumulative reintervention-free survival subdivided for group A (follow-up ≥ 30 years) and group B (follow-up < 30 years). o = reintervention; | = maximal follow-up duration of single patient.

During follow-up, of the 259 patients who survived the arterial switch operation, 65 (25.1%) patients required 105 late reinterventions, with most reinterventions being right-sided (Fig. 1, Fig. 2). For the entire cohort, reintervention-free survival of the arterial switch operation survivors at 10, 20 and 30 years was 83.0%, 76.0% and 76.0% respectively. In log-rank event-free survival analysis, patients from group A were at a greater risk for reintervention than patients from group B (p = 0.005, Fig. 3).

**Fig. 4:**
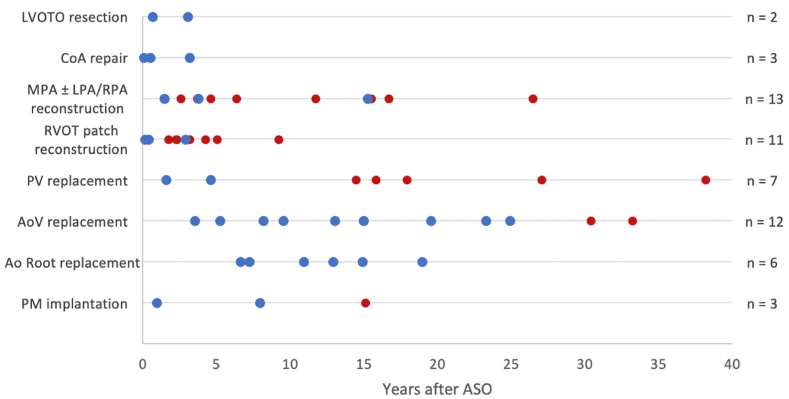
Surgical reinterventions Group A (follow-up ≥ 30 years) = red; Group B (follow-up < 30 years) = blue Abbreviations: LVOTO – left ventricular outflow tract obstruction; CoA – coarctation of the aorta; MPA – main pulmonary artery; LPA – left pulmonary artery; RPA – right pulmonary artery; RVOT – right ventricular outflow tract; PV – pulmonary valve; AoV – aortic valve; PM – pacemaker. (For interpretation of the references to colour in this figure legend, the reader is referred to the Web version of this article.)

**Fig. 5:**
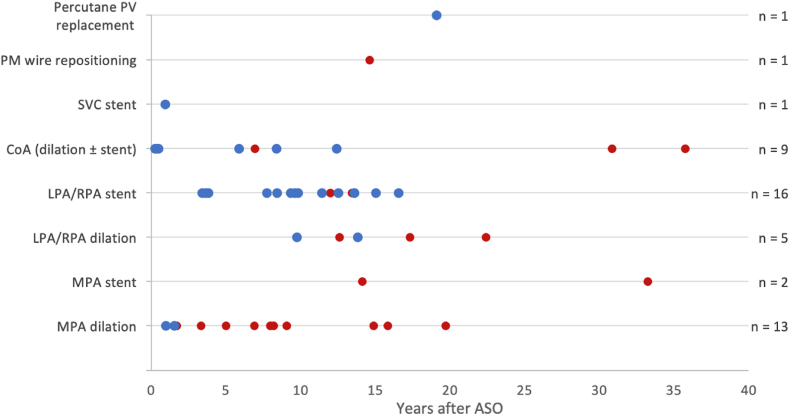
Catheter-based reinterventions Group A (follow-up ≥ 30 years) = red; Group B (follow-up < 30 years) = blue Abbreviations: PV – pulmonary valve; PM – pacemaker; SVC – superior vena cava; CoA – coarctation of the aorta; LPA – left pulmonary artery; RPA – right pulmonary artery; MPA – main pulmonary artery. (For interpretation of the references to colour in this figure legend, the reader is referred to the Web version of this article.)

**Fig. 6:**
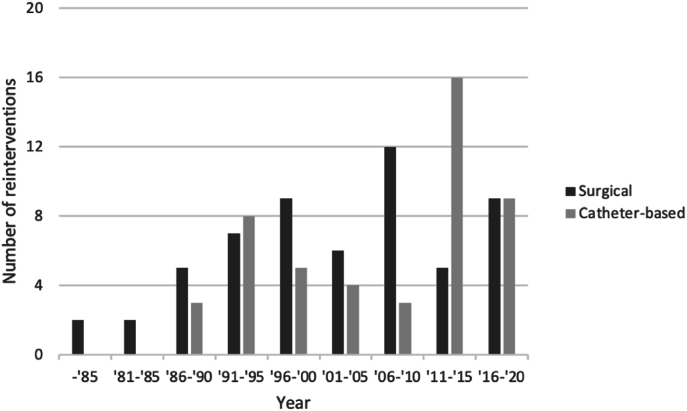
Surgical and catheter-based reinterventions over time.

Surgical reinterventions are shown in Fig. 4, catheter reinterventions are shown in Fig. 5. Fig. 6 depicts the prevalence of surgical (mean date January 31, 2003, IQR = 17.4 years) and catheter-based (mean date May 26, 2011, IQR 18.2 years; p = 0.031) reinterventions over time.

In total, ten patients underwent surgical AoV replacement, of which two patients underwent repeated AoV prothesis replacement. Six patients underwent AoV replacement in their first two decades for AoV regurgitation. Two AoV replacements took place in the third decade of which one for newly developed regurgitation. In the fourth decade, two patients with a late-onset AoV regurgitation needed AoV replacement as their first reintervention.

### 3.5 Predictors for reintervention

In univariate analysis, years since ASO (HR = 1.038, p = 0.007), age at operation (in years)(HR = 1.003, p = 0.006), presence of any early postoperative complication (HR = 1.910, p = 0.03), a complex TGA (HR = 1.714, p = 0.04), and an aortic arch anomaly (HR = 4.865, p < 0.001) predicted risk for reintervention (Table 3). In a multivariate model, only the age at operation (HR = 1.005, p = 0.03), presence of any early postoperative complication (HR = 2.825, p = 0.002), and an aortic arch anomaly (HR = 6.157, p < 0.001) were significant.

#### 4.5.1 Aortic arch anomaly

Reinterventions were more frequent in patients with concomitant arch surgery (66.7% versus 20.6%, p < 0.001) and addressed both the arch as well as the neo-aortic and pulmonary valve. Eight (38.1%) patients in the group with an aortic arch anomaly required a reintervention for re-coarctation of the aorta.

### 3.6 Echocardiography

Through echocardiographic evaluation, group A was compared to the adults in group B. In group A, the LVEF (%) at the patient's last visit was on average low within the normal reference values (53.4 ± 7.6%) (reference values 53–73%), but not significantly lower than the LVEF in adult patients in group B (57.3 ± 8.2%, p = 0.11) (Table 4). Further analysis did not show an association between late LV dysfunction and coronary artery anatomy (t = 1.179, p = 0.241, CI 95% −1.461-5.748). The average LVEDd was normal for the adults in both groups; five patients in group A and two patients in group B eight had a dilated LV (reference values 3.8–5.8 EDd cm). In five patients (four from group A, one from group B) LV dilatation was associated with at least moderate aortic valve regurgitation. The interventricular septum was thicker in group A than in group B (0.9 ± 0.2 cm vs 0.8 ± 0.1 cm, p = 0.02).

**Table 4:** Echocardiographic outcomes adults.

Parameter	Group A	Group B	p-value	Reference values [16,17]
LVEF (biplane Simpson), %, n	53.4 ± 7.6, 17	57.3 ± 8.2, 38^a^	0.11^a^	53–73
LVEDd, cm, n	5.2 ± 0.9, 35	5.0 ± 0.5, 52^a^	0.28^a^	3.8–5.8
IVSd, cm, n	0.9 ± 0.2, 33	0.8 ± 0.1, 51^a^	**0.02**^a^	0.6–0.9
TAPSE, mm, n	19.5 ± 3.8, 40	18.7 ± 3.3, 54^a^	0.25^a^	15–20
E/A ratio, n	**1.9 ± 0.6, 34**	2.3 ± 0.8, 51^a^	**0.005**^a^	≤0.8 & ≥2
Abnormal E/A, n (%)	19/34 (55.9)	15/50 (30.0)	**0.02**^a^	n/a
Mitral E/e’ ratio (IQR, n)	6.8 (5.4–7.5, 28)	6.6 (5.2–7.7, 34)^a^	0.83^a^	≤14
Septal e’, n	10.9 ± 2.4, 23	11.3 ± 3.4, 27^a^	0.65^a^	≥7
Maximal aortic annulus diameter, cm, n	**3.8 ± 0.7, 29**	**3.6 ± 0.5, 45**^a^	0.11^a^	2.9–3.5
Dilated, n (%)	19/29 (65.5)	27/44 (61.4)	0.72^a^	n/a
≥ moderate TR, N/n (%)^b^	3/44 (6.8)	4/161 (2.5)	0.16	n/a
≥ moderate PR, N/n (%)^b^	1/29 (3.4)	4/152 (2.6)	0.81	n/a
≥ moderate AR, N/n (%)^b^	3/39 (7.7)	8/153 (5.2)	0.56	n/a

Abbreviations: LV – Left ventricular; EF – Ejection fraction; n – number of patients in group with parameter measurement; EDd – End diastolic diameter; IVSd – Interventricular septal end systolic diameter; TAPSE – Tricuspid annular plane systolic excursion; MV E/A ratio – Mitral valve early diastolic velocity divided by atrial contraction velocity; Mitral E/e’ ratio – MV E divided by mitral annular early diastolic velocity; Septal e’ – Septal annular early diastolic velocity; PV – Pulmonary valve; TR – Tricuspid regurgitation; PR – Pulmonary regurgitation; AR – Aortic regurgitation; N/a – Not available.

Bold refers to statistically significant p-values.

a Only includes patients ≥18 years of age, N = 81.

b Patients with valve replacement excluded.

In group A, an abnormal E/A ratio was more frequent than in group B (55.9 vs 30.0%). The mitral septal E/e’ ratio and septal e’ were normal for both groups (reference values ≤ 14 and ≥ 7 cm/s respectively).

The maximal aortic root diameter was dilated on average in both group A (3.8 ± 0.7 cm) and group B (3.6 ± 0.5 cm). In total 44 survivors had a maximal aortic root diameter of more than 3.5 cm (reference values 2.9–3.5 cm) [19], of which 19 in group A and 25 in group B.

In both groups, aortic regurgitation (A: 7.7%, B: 5.2%) was more prevalent than pulmonary (A: 3.4%, B: 2.6%) and tricuspid (A: 6.8%, B: 2.5%) regurgitation. There was no significant difference between group A and B.

For the pediatric cohort (age <18 years) the following z-scores were available: mean LVEDd 0.06 ± 1.1% (min-max: -3.20 – 4.00, n = 92), mean IVSd −0.20 ± 0.78 cm (min-max: -1.90 – 1.30, n = 90), maximal aortic root diameter 2.75 ± 1.36 (min-max: -1.50 – 5.50, n = 77).

### 3.7 Functional outcome

Based on clinical follow up data, mildly restricted exercise capacity (NYHA II) was described in 4 group A patients and 1 group B patient (p < 0.01). There were no patients in NYHA III or IV. Eight patients were on medication to reduce afterload with impaired LV function (4) and to treat paroxysmal SVT (4).

## 4 Discussion

### 4.1 Study population and mortality

We described a single center cohort of arterial switch patients which includes a relatively large number (59) of patients with extended follow up who were operated before 1991. This specific cohort allowed us to evaluate clinical outcomes beyond 30 years after operation.

First, the majority of patients who survived the pioneer arterial switch operations in the era before 1986, are typically doing clinically well today. Five of 254 long-term survivors had reduced functional class and eight patients were dependent on cardiac medication of which the majority in group A. Early outcome in terms of operative mortality improved significantly over the studied period. This is well in agreement with the trend in declining discharge mortality of other STAT 3 and 4 operations as reported by Jacobs et al. [20], who analyzed the mortality trends in pediatric and congenital heart surgery according to the classification of the Society of Thoracic Surgeons-European Association for Cardio-Thoracic Surgery (STAT). In the most recently operated group of patients, the number of peri-operative complications was higher compared to the older cohort. When perioperative mortality and perioperative reinterventions are combined, comparable levels between group A and B are reached. Possibly, survival upon a peri-operative complication has improved over time, reducing the peri-operative mortality due to peri-operative complications. Another likely explanation for the higher number of peri-operative complications in the younger cohort is the improved complication registration. Late mortality was unusual in both groups and was related to a cardiac cause in two of the five patients.

### 4.2 Reinterventions

In contrast, cardiac reinterventions for residual lesions were relatively frequent in the

Entire group. Most of these being indicated for right sided lesions, as reported previously [[6], [7], [8], [9],[21], [22], [23]]. Reinterventions were initially mainly surgical; however, shifted to catheter-based in the recent era. Although we expected pulmonary reinterventions to be related to previous pulmonary artery banding or early corrections without Lecompte maneuver, this was not confirmed in our risk analysis. Moreover, the majority of reinterventions were performed in the first 20 years after ASO.

Most reinterventions were performed in the oldest cohort patient group A, reflected in the Kaplan Meier curve of patients group A compared to group B (p = 0.005). Furthermore, the presence of any early postoperative complication is a strong predictor of reinterventions HR = 2.825, p = 0.002). Additionally, an aortic arch anomaly was identified as a risk factor for reintervention. This was also found by the long-term follow-up study by Van der Palen et al. [9]. Similarly, Fricke et al. demonstrate that patients diagnosed with TGA associated with aortic arch obstruction (AAO) have a significantly higher reoperation rate than TGA without AAO [24]. Moreover, they conclude that patients with TGA and AAO were more likely to need additional right-sided reinterventions than TGA patients without AAO. In TGA patients with AAO the native aortic annulus is often relatively small or even with subvalvular obstruction, possibly causing these patients to be at a higher risk of right-sided obstruction [24]. Reintervention in TGA with associated arch anomaly is well known; however, the rate of left-sided reinterventions after correction of TGA with aortic arch anomaly is comparable to reinterventions for isolated coarctation repair (38% vs 31%) [25]. This percentage may be high due to an early operation age (≤1 year). In contrast to Van der Palen et al. [9], in this cohort coronary artery anatomy and a complex TGA were not found to be independent risk factors for reintervention.

Whereas left sided reinterventions occurred less often, it is of note that in fourth decade of follow-up left sided reinterventions were seen more frequently than right sided reinterventions. In this decade two patients, who did not need a reintervention before, required AV replacement. Of note, echocardiographic data showed a dilated aortic annulus, also identified after follow-up of the aortic root post ASO by Van der Palen et al. [26]. This might indicate that more AV replacements may be necessary in the decades of follow-up ahead due to long-lasting aortic valve dilation, which is in accordance with a long-term follow-up study by Fricke et al. [27]. In their study, the 95 patients who were more than 25 years after ASO, 15% had at least moderate aortic regurgitation or had undergone neoaortic valve replacement.

### 4.3 Echocardiographic and functional follow-up

Perhaps most notably in our study we observed low normal LVEF and difference in E/A ratio between group A and B.

In patients from group A, LV function estimated by LVEF was just within normal reference values; however, it trended to be lower than the LVEF in group B. A recent meta-analysis also found a normal LVEF after ASO (60.7 ± 7.2%) at an average follow-up of 17.4 years [28]. Nonetheless, the LVEF in group A of our study is significantly lower when compared to the results of this meta-analysis, with a mean difference of 7.3% (p < 0.001, CI95% 3.84–10.76). This may be explained by a significantly longer follow-up period in group A than the patients in this meta-analysis (34.8 vs 17.4 years). Reduced LVEF in comparison to healthy control subjects had been reported by others [29,30]. Furthermore, Di Salvo et al. found subclinical dysfunction in a decreased global longitudinal strain [31], and Grotenhuis et al. found signs of diffuse myocardial fibrosis through CMR T1 mapping [32]. Moreover, Pettersen et al. investigated ventricular function after ASO and found a subclinical myocardial dysfunction characterized by a decreased global LV longitudinal strain and decreased LV torsion [33]. This study did, however, describe a normal LVEF (57 ± 5%) in a cohort of 22 patients with 12.4 years follow-up. They speculate that a subclinical myocardial dysfunction could develop into left ventricular disfunction later on, as demonstrated in our study [33]. With this in mind, continuous clinical follow-up including LVEF is warranted, particularly long-term after ASO. A study with a much shorter follow-up (3.5 years) evaluated echocardiographic function in which patients who underwent pulmonary artery banding were isolated [34]. This study found that pulmonary artery banding resulted in non-progressive depressed contractility, abnormal wall motion, myocardial perfusion abnormalities, and reduced EF. Serial evaluations showed no relation between left ventricular dysfunction and longer follow-up. The short follow-up time of this study makes this relation difficult to compare to the current cohort; however, it is important to note, since in group A significantly more patients underwent pulmonary artery banding [34].

As for diastolic dysfunction, an aberrant E/A ratio was found more frequently in group A. However, the American Society of Echocardiography and the European Association of Cardiovascular Imaging guidelines recommend using the E/e’ ratio and e’ velocity over the E/A ratio [18]. When isolating E/e’ ratio and e’ velocity the cohort showed no clear diastolic dysfunction. A follow-up study by Pettersen et al. (n = 22, follow up 12.4 ± 2.3 years) found a normal diastolic function [33]. However, Xie et al. did find a higher E/e’ in patients after ASO compared to healthy controls [35]. Both Pettersen and Xie address that decreased LV torsion could result in lower diastolic lengthening velocity and thus impaired LV relaxation, resulting in increased LV filling pressure and a higher E/e’ [33,35]. A decrease in diastolic function related to age has also been described in literature [36]. However, Wang et al. suggests that this relation might be most apparent from an age of 50 years [37]. This would imply a markedly stronger decrease in LV diastolic function over time in our patient cohort.

In addition to the low-normal LVEF values which are significantly lower than the findings of a recent meta-analysis [28], indications in literature that the left ventricle is not functioning optimally, and one abnormal diastolic function parameter, more patients in group A showed clinical signs of ventricular dysfunction (4 vs 1 in NHYA class II).

### 4.4 Limitations

This study is subject to inherent limitations by its retrospective nature. Moreover, while accounted for in follow-up time, patients did not undergo standardized post ASO evaluation with routine imaging and exercise testing.

## 5 Conclusion

The majority of patients in their fourth decade after the arterial switch operation are in functional class I. Early survival has improved over time, whereas late mortality is rare. The need for reintervention is decreasing over time, whereas catheter-based has become the preferential mode of reintervention. A trend towards reduced LV function and late aortic valve replacements justify further research and warrant follow-up.

## Sources of funding

None.

## Declaration of competing interest

The authors declare that they have no known competing financial interests or personal relationships that could have appeared to influence the work reported in this paper.
